# DNA damage accumulation and TRF2 degradation in atypical Werner syndrome fibroblasts with LMNA mutations

**DOI:** 10.3389/fgene.2013.00129

**Published:** 2013-07-05

**Authors:** Bidisha Saha, Galynn Zitnik, Simon Johnson, Quyen Nguyen, Rosa A. Risques, George M. Martin, Junko Oshima

**Affiliations:** Department of Pathology, University of WashingtonSeattle, WA, USA

**Keywords:** lamin A/C, telomeres, TRF2, progeroid syndromes, laminopathies

## Abstract

Segmental progeroid syndromes are groups of disorders with multiple features suggestive of accelerated aging. One subset of adult-onset progeroid syndromes, referred to as atypical Werner syndrome, is caused by mutations in the *LMNA* gene, which encodes a class of nuclear intermediate filaments, lamin A/C. We previously described rapid telomere attrition and accelerated replicative senescence in cultured fibroblasts overexpressing mutant lamin A. In this study, we investigated the cellular phenotypes associated with accelerated telomere shortening in *LMNA* mutant primary fibroblasts. In early passage primary fibroblasts with R133L or L140R *LMNA *mutations, shelterin protein components were already reduced while cells still retained telomere lengths comparable to those of controls. There was a significant inverse correlation between the degree of abnormal nuclear morphology and the level of TRF2, a shelterin subunit, suggesting a potential causal relationship. Stabilization of the telomeres via the introduction of the catalytic subunit of human telomerase, hTERT (human telomerase reverse transcriptase), did not prevent degradation of shelterin components, indicating that reduced TRF2 in *LMNA* mutants is not mediated by short telomeres. Interestingly, γ-H2AX foci (reflecting double strand DNA damage) in early passage *LMNA* mutant primary fibroblasts and *LMNA* mutant hTERT fibroblasts were markedly increased in non-telomeric regions of DNA. Our results raise the possibility that mutant lamin A/C causes global genomic instability with accumulation of non-telomeric DNA damage as an early event, followed by TRF2 degradation and telomere shortening.

## INTRODUCTION

Segmental progeroid syndromes are a genetically heterogeneous collection of disorders whose presentations resemble accelerated aging (Martin, 1978). The best known example of an adult-onset segmental progeroid syndrome is the Werner syndrome (“Progeria of Adult”; WS), caused by mutations in the *WRN* gene (Yu et al., 1996; Friedrich et al., 2010). Our International Registry of Werner syndrome () has accumulated several dozen cases of “atypical” WS (AWS) – cases submitted by clinicians to us as examples of WS, but lack *WRN* mutations and have normal levels of the WRN protein. A small subset of AWS cases are caused by *LMNA* mutations (Chen et al., 2003). The *LMNA* gene encodes nuclear intermediate filaments, lamin A and lamin C, the major components of the nuclear lamina. These provide the nucleus with mechanical support and contribute to functional interactions with chromatin (Broers et al., 2006; Andres and Gonzalez, 2009). R133L and L140R are heterozygous dominant *LMNA *mutations located at the surface of a heptad repeat, suggesting that these mutations might perturb the intermolecular interactions of lamin A/C (Chen et al., 2003). There have been additional reports of heterozygous dominant *LMNA* mutations in AWS cases, including our recent report of heterozygous substitutions at the junction of *LMNA* exon 11 and intron 11, which results in the generation of a very small amount of progerin (Hisama et al., 2011). Progerin is an aberrant splice form of lamin A; high levels are responsible for Hutchinson–Gilford progeria syndrome (“Progeria of Childhood”; HGPS; De Sandre-Giovannoli et al., 2003; Eriksson et al., 2003). In addition to progeroid syndromes, *LMNA* mutations are also responsible for muscular dystrophies, cardiomyopathies, partial lipodystrophies, and peripheral neuropathies (Broers et al., 2006).

Cells carrying *LMNA* mutations exhibit characteristic morphological nuclear abnormalities and altered mechanical properties of the lamina (Dahl et al., 2006). In addition to its structural roles, the nuclear lamina interacts with chromatin to participate in chromatin remodeling and heterochromatin organization, providing maintenance of nuclear organization, (Broers et al., 2006; Andres and Gonzalez, 2009), and thus influencing replication timing and transcription (Gonzalez-Suarez and Gonzalo, 2010; Hansen et al., 2010). In HGPS fibroblasts, changes in the lamina structure result in reorganization of heterochromatin and epigenetic changes in histones (Shumaker et al., 2006).

Cellular characteristics of human fibroblasts expressing progerin include genome instability such as increased γ-H2AX foci, continuous p53 activation (Liu et al., 2005; Scaffidi and Misteli, 2006; Kudlow et al., 2008; Musich and Zou, 2011), and defective cell proliferation, which can be partially overcome by inactivation of p53, ATM (ataxia telangiectasia mutated), and ATR (ATM- and RAD3-related) or introduction of human telomerase reverse transcriptase (hTERT; Liu et al., 2006; Kudlow et al., 2008). Furthermore, several studies have shown a role for A-type lamins in telomere maintenance (Gonzalez-Suarez et al., 2009; Benson et al., 2010). In primary fibroblasts derived from HGPS patients, DNA damage foci were shown to be localized to the telomeres in association with accelerated replicative senescence (Benson et al., 2010). hTERT immortalization of HGPS fibroblasts has been shown to lead to a reduction of p53 pathway signaling, further supporting the idea that the telomere may be a major DNA target of progerin (Benson et al., 2010). However, in an independent study, hTERT failed to prevent the accumulation of DNA damage upon induction of exogenous progerin expression (Scaffidi and Misteli, 2008). Studies of *LMNA* knockout mice provided evidence that lamin A/C interacts with telomeres and maintains the distribution of telomeres throughout the nucleus (Gonzalez-Suarez et al., 2009). Altered distributions of telomeres in *LMNA* knockout mouse cells were associated with deprotection and hyper-recombination of telomeric DNA, resulting in accelerated telomere loss and a DNA damage response (Gonzalez-Suarez et al., 2009). This suggests that, depending on the nature of the *LMNA* mutation(s), altered lamin A/C may lead to accelerated telomere shortening via different mechanisms.

We previously demonstrated that human fibroblasts which over-express either the wild type lamin A, the mutant lamin A found in HGPS patients (delta 35 and delta 50), or the lamin A mutations in AWS patients (R133L and L140R), all undergo accelerated telomere attrition and rapid replicative senescence (Huang et al., 2008). Unlike the classical deletions that cause HGPS, amino acid substitutions of lamin A/C, such as those found in patients with AWS, have not been well studied for their effects on telomeres. It has been well established that telomeres become dysfunctional through replicative telomere attrition, inhibition of shelterin or the activation of oncogenes (Denchi and de Lange, 2007; Sfeir and de Lange, 2012; Suram et al., 2012). In this study, we investigated the cellular phenotypes associated with accelerated telomere shortening in AWS fibroblasts focusing on the shelterin complex. The shelterin complex protects chromosomal ends from erosions and consists of six major proteins, TRF2, TRF1, POT1, TPP1, Rap1, and TIN2 (Palm and de Lange, 2008). The POT1–TPP1 complex competes with the CST (CTC1, STN1, and TEN1) complex which terminates telomerase activity (Chen et al., 2012). Among shelterin components, TRF2 in particular is thought to play an important role in the protection of telomeres from erosion by binding to telomeric repeats to form a t-loop (Palm and de Lange, 2008). Loss of TRF2 protein expression or activity was found to trigger telomere shortening, the DNA damage response, chromosomal instability, and replicative senescence (Oh et al., 2003; Denchi and de Lange, 2007; Okamoto et al., 2013). TRF2 was shown to co-precipitate with lamin A (Luderus et al., 1996) and also to interact with lamin-associated proteins, LAP-2α and BAF, in a cell cycle dependent manner (Dechat et al., 2004). In the present study we observed significantly reduced levels of TRF2 and other shelterin proteins in *LMNA* mutant AWS cells. We suggest that this reduction in protein levels is due to the degradation of TRF2 protein. Using immuno-fluorescent *in situ* hybridization (immuno-FISH) analysis we found that *LMNA* mutant cells accumulate increased levels of non-telomeric DNA damage in *LMNA* mutant hTERT cells. We therefore suggest that these forms of mutant lamin A induce genomic DNA damage and that such damage may contribute to the reduction of TRF2 proteins via activation of the p53-mediated signaling cascades, leading to the degradation of shelterin proteins. Suboptimum shelterin-telomere interactions may further lead to a loss of protection of telomeric ends and subsequent telomere attrition.

## MATERIALS AND METHODS

### ETHICS STATEMENT

Patients signed approved consent forms to participate in the studies of the International Registry of Werner Syndrome (Seattle, WA, USA) prior to the initiation of the study. This study was approved by the Human Subjects Division, Institutional Review Board of the University of Washington, Seattle, WA, USA.

### CELL LINES AND CELL CULTURE

Human diploid fibroblast cell lines were established from biopsies of skin samples. Cell lines 82-6 and 88-1 (normal) were obtained from newborn foreskin and the abdominal skin of a 62 year old female, respectively. Fibroblasts were cultured in Dulbecco’s modified Eagle’s medium (DMEM; Invitrogen, Carlsbad, CA, USA) supplemented with 10% heat-inactivated fetal bovine serum (FBS; Thermo Scientific Hyclone, OH, USA) and penicillin and streptomycin (Invitrogen), as previously described (Chen et al., 2003). The media was changed every 3–4 days. All cells were cultured at 37°C in 5% CO_2_. Primary fibroblasts were immortalized with a construct encoding hTERT (Rubio et al., 2002). MG-132 (10 μM, Calbiochem, La Jolla, CA, USA) and cycloheximide (100 μg/ml, Sigma, St. Louis, MO, USA) was used when treating fibroblasts.

### BrdU INCORPORATION ASSAY

To determine the fraction of proliferating cells, a 5-bromo-2′-deoxyuridine (BrdU) incorporation assay was carried out using a BrdU labeling and detection kit (11 296 736 001, Roche Applied Science, Indianapolis, IN, USA) according to the manufacturer’s instructions. Briefly, primary fibroblasts from control, *LMNA* mutant and *WRN* mutant cell lines were grown for 24 h on cover slips. Cells were then labeled with BrdU (10 μM) for 24 or 48 h, fixed in 70% ethanol with 50 mM glycine for 20 min at -20°C and processed for immunofluorescence. Cells were then incubated with the primary antibody, anti-BrdU (mouse monoclonal antibody, clone BMG 6H8 IgG1, 1:10 dilution) for 30 min at 37°C followed by Alexa Fluor 594-conjugated goat anti-mouse antibody (1:200 dilution; A-11005, Molecular Probes, Eugene, OR, USA) for 30 min at 37°C. Cells were mounted onto slides using Vectashield with DAPI (Vectashield, Burlingame, CA, USA) and observed and photographed with Nikon Upright (Nikon Eclipse E600) microscope at the Keck Center for Imaging, University of Washington, Seattle, WA, USA. The percentages of cells with BrdU-labeled nuclei were determined by counting 300 cells per data point.

### WESTERN BLOT ANALYSIS

For western blot analyses, cells were lysed in 2× Laemmli buffer [0.5 M Tris pH 6.8, 10% sodium dodecyl sulfate (SDS) glycerol]. Fifteen micrograms of total protein isolated from primary fibroblasts or immortalized fibroblast cell lines were separated on NuPAGE 4–12% gradient Bis–Tris gel (Invitrogen) and transferred to polyvinylidene fluoride (PVDF) membranes. The membranes were incubated with anti-TRF2 (IMG-124A, clone 4a794.15, Imgenex, San Diego, CA, USA and sc-32106, Santa Cruz Biotechnology Inc., Santa Cruz, CA, USA), anti-TRF1 (sc-5596, Santa Cruz Biotechnology Inc), anti-POT1 (sc-27952, Santa Cruz Biotechnology Inc.), anti-TIN2 (IMG-282, clone 59B388.1, Imgenex), anti-hRap1 (IMG-289, Imgenex), anti-TPP1 (ab54685, Abcam Inc., Cambridge, MA, USA) at a dilution of 1:2000 or anti-β-actin (clone AC-15, Sigma, St Louis, MO, USA) at a dilution of 1:40,000 in a milk blocking solution, then with corresponding biotinylated secondary antibodies against mouse, rabbit or goat IgG (Vector Laboratories, Burlingame, CA, USA). The reactions were visualized with western lightening chemiluminescence reagent (NEL100, Perkin Elmer, Waltham, MA, USA) according to the manufacturer’s instruction. The bands corresponding to TRF2, TRF1, POT1, TIN2, hRap1, and TPP1 were quantified using TotalLab software (Newcastle, UK) and normalized against β-actin levels.

For the measurement of the TRF2 protein half-life, immortalized fibroblast cell lines from the control, *LMNA* mutant and *WRN *mutant were treated with cycloheximide (100 μg/ml) for 8–12 h. Total protein extracts were subjected to the western analysis of TRF2 and β-actin as described above. After normalizing to β-actin levels, TRF2 levels were calculated in order to estimate the protein half-lives.

### QUANTITATIVE REAL TIME PCR

Quantitative reverse transcription polymerase chain reaction (RT-PCR) for *TRF2* mRNA was performed using Taqman gene expression assay system (Hs00194619_m1, Applied Biosystem, Foster City, CA, USA) and normalized against mRNA level of GAPDH (Hs02758991_g1, Applied Biosystem). Total RNA was isolated from the cultured cell pellet using Qiagen RNA isolation kit (Qiagen, Valencia, CA, USA) following the manufacturer’s instructions. Reverse transcription was carried out with 2 μg isolated total RNA. Quantitative PCR was performed with a Rotor-Gene 3000 (Corbett Research, Sydney, Australia). The data analysis was performed as previously described (Huang et al., 2008).

### TELOMERE QUANTITATIVE POLYMERASE CHAIN REACTION

Mean telomere lengths were determined by qPCR as previously described (Huang et al., 2008). Genomic DNA was isolated via a salting out method and qPCR was performed with a Rotor-Gene 3000 (Corbett Research, Sydney, Australia). The data were analyzed as previously described (Huang et al., 2008). The intra-assay and inter-assay variability (CV) for qPCR was 3 and 5%, respectively.

### TELOMERASE REPEAT AMPLIFICATION PROTOCOL ASSAY

Telomerase activity in hTERT-immortalized cell lines was measured using the TRAPEZE XL Telomerase detection kit (S7707, Millepore, Billerica, MA, USA). Cells were lysed in ice-cold 3-[(3-cholamidopropyl)dimethylammonio]-1-propane-sulfonate (CHAPS) lysis buffer for 30 min. 2 μl of the cell extract was analyzed for the telomerase repeat amplification protocol (TRAP) assay. In the first step telomeric repeats were added to a substrate oligonucleotide by telomerase; then, the extended products were amplified by Taq polymerase using PCR with TS and RP (reverse) primers, generating a ladder of products in 6 base increments. The PCR products were separated on a 10% non-denaturing polyacrylamide gel (PAGE). The gel was stained with SYBR Safe (S33102, Invitrogen) to visualize the TRAP ladder using FluorChem Q imaging system (Alpha Innotech, San Leandro, CA, USA).

### IMMUNO-FISH

Immuno-fluorescent *in situ* hybridization was performed as described previously (O’Sullivan et al., 2004) with modifications. Cells were grown on coverslips overnight and then fixed the next day with 3.7% paraformaldehyde in phosphate buffered saline (PBS; pH 7.4) plus 0.2% Triton X100 for 10 min at room temperature. Cells were blocked for 30 min at 37°C and incubated with primary antibody, mouse anti-TRF2 (IMG-124A, clone 4a794.15, Imgenex) or rabbit anti-γ-H2AX (2577, Cell Signaling Technologies, Beverly, MA, USA) for 30 min at 37°C. They were then incubated with secondary antibody, donkey anti-mouse Alexa Fluor or donkey anti-rabbit Alexa Fluor (Invitrogen Molecular Probes, Eugene, OR, USA) for 1 h at room temperature, and then post-fixed with 3.7% paraformaldehyde for 5 min. After washing in PBS, cells were processed for FISH. Cells were treated with 100 μg/ml of RNase A (Sigma-Aldrich, St. Louise, MO, USA) in PBS for 45 min. at 37°C, then dehydrated in an ethanol series and air dried. Peptide nucleic acid (PNA) probe (200 nM, Cent-FAM, TelC-FITC, or TelC-Cy3; Panagene Inc., Daejeon, Korea) in hybridization buffer was placed on top of the coverslips, heated to 75–78°C for 15 min, then removed, wrapped in foil and allowed to sit at room temperature overnight. The next day the coverslips were washed with 70% formamide in Tris buffer, washed with preheated (55–60°C) Tween 20 solution, dehydrated in an ethanol series and then air dried. Coverslips were mounted on slides with DAPI (Vectashield) and sealed with clear nail polish.

### IMAGE PROCESSING AND ANALYSIS OF TELOMERE LENGTH, TRF2 INTENSITIES AND CO-LOCALIZATION

The images were obtained in Z-series with Deltavision deconvolution microscope (Applied Precision, Issaquah, WA, USA) at the Keck Center (University of Washington, Seattle, WA, USA). The images were deconvolved using SoftWorx software (Applied Precision, Issaquah, WA, USA). They were analyzed for average telomere intensity (which is proportional to telomere length), and average TRF2 intensity using a software program, 4xColocalization, version 138, provided by the University of Washington Nathan Shock Center, as described previously (O’Sullivan et al., 2004). The images were also analyzed for co-localization (telomere and TRF2; telomere and γ-H2AX; TRF2 and γ-H2AX). Statistical significance was determined by the Student *t*-test (Chen et al., 2003; Huang et al., 2008).

### NUCLEAR MORPHOLOGY AND CORRELATION ANALYSIS

To assess the degree of nuclear irregularity, nuclear contour ratios (NCRs) were determined in randomly selected nuclei of *LMNA *mutant fibroblasts using MetaMorph software as described previously (Saha et al., 2010). The nuclei were also analyzed for average TRF2 intensities. The correlation between NCR and TRF2 intensities was analyzed using Pearson’s correlation coefficient, *r*. For all experiments, a two-tailed *P *value of <0.05 was considered significant.

## RESULTS

### REDUCTION OF SHELTERIN COMPONENTS IN PRIMARY *LMNA* MUTANT FIBROBLASTS

We first analyzed the steady state protein expression levels of the shelterin subunits TRF2, TRF1, POT1, TIN2, TPP1, and Rap1 in primary fibroblasts carrying a heterozygous R133L mutation derived from Registry# PORTU8010 and in primary fibroblasts carrying a heterozygous L140R mutation derived from Registry# NORWAY1010 (Chen et al., 2003). The controls were two normal primary fibroblast cultures, 82-6 and 88-1 (Oshima et al., 1995; Huang et al., 2008) as well as cultures of *WRN* mutant fibroblasts, Registry# MCI7885, derived from a patient with classical Werner syndrome (Oshima et al., 1995; Yu et al., 1996). The latter was included as a “control” since these fibroblasts undergo accelerated telomere shortening by different mechanisms, namely defects in lagging strand synthesis of telomeres (Crabbe et al., 2004). We examined fibroblasts in early passages (BrdU labeling index higher than 60%) in order to detect proximal changes rather than changes secondary to telomere shortening and replicative senescence.

**FIGURE 1:**
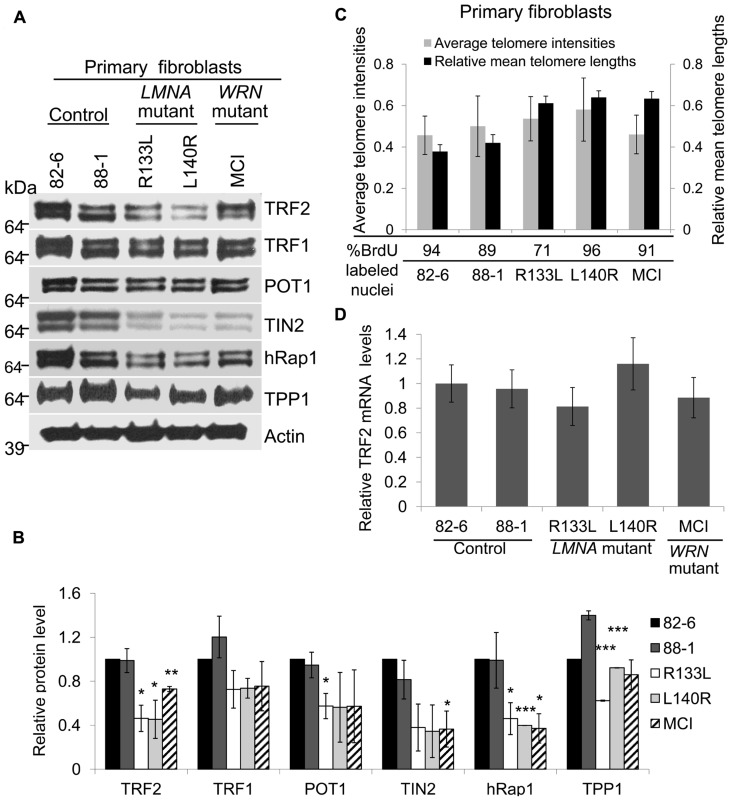
** Expression of shelterin subunits in *LMNA* mutant primary fibroblasts.** **(A)** Western analysis of TRF2, TRF1, POT1, TIN2, hRap1, and TPP1 in control, *LMNA* and *WRN* mutant primary fibroblasts. Protein levels were normalized to β-Actin. Autoradiographies with exposures beyond the linear range are shown for clarification of bands. **(B)** Densitometric scanning of band intensities obtained from western analysis. Data are means ± s.d. from three independent experiments. *P < 0.05; **P < 0.01; ***P < 0.001. **(C)** Analysis of relative telomere lengths expressed as average telomere intensities ± s.d. in indicated cell types of control, *LMNA* mutant and *WRN* mutant fibroblasts. The signal intensities were analyzed by Q-FISH using a telomere-specific peptide nucleic acid (PNA) probe. A centromere PNA probe was used as an internal standard. Approximately 70 cells were analyzed for each cell line. The relative mean telomere lengths (±s.d.) in fibroblasts were measured by quantitative PCR. **(D)** *TRF2* mRNA expression in normal, *LMNA *mutant and *WRN* mutant fibroblasts. *TRF2* mRNA expression was detected by qRT-PCR. *GAPDH* mRNA expression was used as an internal standard. Data are presented as mean ± s.d. from three independent experiments. Control (82-6) values were taken as onefold.

Western analysis showed that most subunits of the shelterin complex were reduced in both types of *LMNA* mutant fibroblasts (**Figure 1A**). Steady state levels of TRF2 were 46 and 45%, POT1 was 57 and 56%, TIN2 was 36 and 35%, and Rap1 was 46 and 40%, in R133L and L140R mutants, respectively, compared to the control, 82-6 fibroblasts (**Figure 1B**). The other two components, TRF1 and TPP1, were also slightly reduced to 73 and 62% in R133L mutants and 74 and 92% in L140R mutants (**Table 1**). Interestingly, *WRN* mutant fibroblasts also showed reduced protein expression of all of the shelterin components.

**Table 1:** Mean values of relative protein levels of shelterin components in primary and hTERT-immortalized fibroblasts of *LMNA* mutant cell lines. Protein levels in mutant lines were normalized to the average in controls, 82-6 and 82-6 hTERT.

Shelterin components	*LMNA* mutant cell lines			
	PORTU8010		NORWAY1010	
	R133L	R133L hTERT	L140R	L140R hTERT
TRF2	0.462	0.544	0.453	0.370
TRF1	0.726	0.429	0.736	0.362
POT1	0.574	0.491	0.562	0.470
TIN2	0.363	0.785	0.345	0.590
hRap1	0.460	0.590	0.398	0.312
TPP1	0.623	1.319	0.921	1.212

Since the length of the telomere determines the number of available binding sites for the shelterin complex, cells with short telomeres (i.e., late passage or senescent cells) would require and presumably express lower amounts of the shelterin components. To rule out this possibility, relative telomere lengths were measured by quantitation of fluorescence *in situ* hybridization images (Q-FISH), as established for normal fibroblasts undergoing replicative senescence (O’Sullivan et al., 2004). Using this method, we found that the telomere lengths of young *LMNA* mutant fibroblast cultures were comparable to those of controls after normalizing to centromere signal intensities (**Figure 1C**). Mean telomere lengths were also measured by quantitative PCR, showing no significant differences among *LMNA* mutant fibroblasts and controls (**Figure 1C**). The mean telomere lengths of the two controls were similar despite the difference in the age of donors, 82-6 (newborn) and 88-1 (adult; **Figure 1C**). This may be due to the fact that there is a wide range of mean telomere lengths and replicative life spans among normal individuals. The labeling index determined by the 48 h BrdU incorporation and population doubling (PD) of the culture were 94% in 82-6 (PD 9), 89% in 88-1 (PD 7), 71% in R133L (PD 5), 96% in L140R (PD 6), and 91% in MCI7885 (PD 6), confirming that the *LMNA* mutant fibroblast cultures used in these experiments were indeed from young passages. This indicates that the reduction of shelterin proteins observed in *LMNA* mutants was not a direct result of shortened telomeres.

Next, we focused on the role of TRF2, a major component of the shelterin complex, in preventing telomere erosion. To determine whether the reduction of TRF2 proteins in *LMNA* mutant fibroblasts occurs via a transcriptional mechanism or post-transcriptional degradation, we analyzed TRF2 expression at the mRNA level. The qRT-PCR results showed that the steady state levels of TRF2 mRNA in *LMNA* mutant fibroblasts were not significantly different when compared to the controls (**Figure 1D**). These results suggest that the TRF2 protein may undergo degradation in *LMNA* mutant cells, resulting in low TRF2 protein levels while the cells still retain long telomeres.

### NUCLEAR MORPHOLOGICAL ABNORMALITIES CORRELATE WITH REDUCTIONS OF TRF2 WITHIN *LMNA* MUTANT CULTURES

**FIGURE 2:**
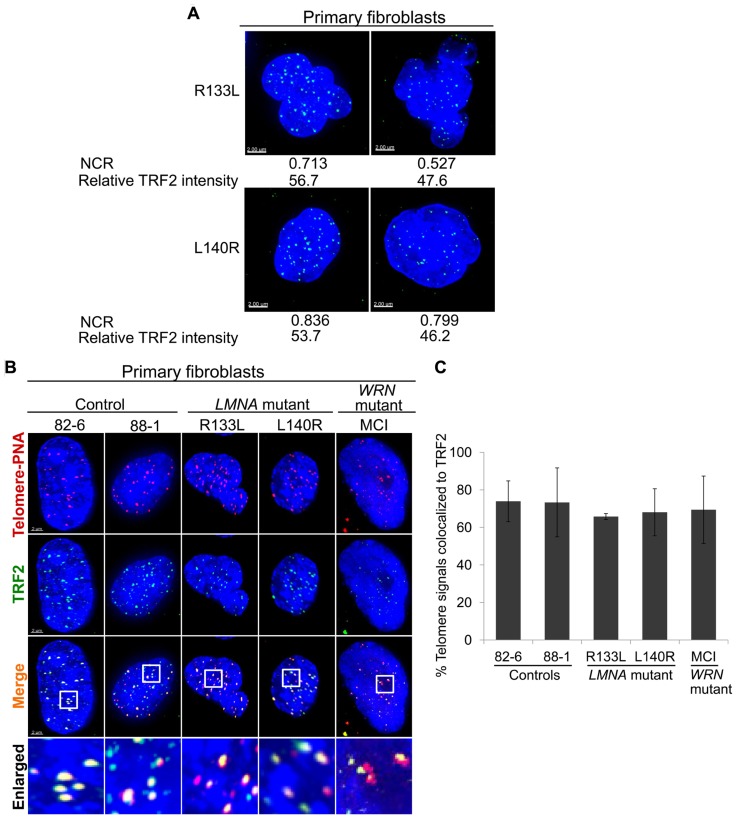
** Intranuclear localization ofTRF2 in primary fibroblasts.** **(A)** Abnormal nuclear morphology as measured via NCR and TRF2 expression (green) in *LMNA* mutant cells. **(B)** Immuno-FISH staining of telomeres (using a telomeric PNA probe; red), TRF2 (green) and DNA (using DAPI; blue) in control, *LMNA* and *WRN* mutant cells. White boxes in the merged images indicate areas that are enlarged 5× and shown to the bottom. Scale bar: 2 μm. **(C)** Fractions of telomeric DNA co-localized to TRF2. Data were presented as means ± s.d. Approximately 70 cells were analyzed for each cell line.

We previously reported that primary *LMNA* mutant fibroblasts, but not control fibroblasts or immortalized *LMNA* mutant cells, exhibit abnormal nuclear morphology, as assessed by NCR (Chen et al., 2003). In control fibroblasts, 82-6, the contour ratio was 0.842 ± 0.036, whereas in R133L the contour ratio was 0.749 ± 0.118 (*P* < 0.005); for L140R, the ratio was 0.815 ± 0.074 (*P *< 0.05). We noted that within the *LMNA* mutant cultures, the cells with more distorted nuclei tended to have lower levels of TRF2 (**Figure 2A**). Quantitative analysis revealed a significant positive correlation between NCR and TRF2 protein level per nucleus in both R133L and L140R mutant cultures: Pearson’s correlation coefficient, *r*, was 0.365 for R133L (*P *< 0.005) and was 0.379 for L140R (*P *< 0.05; **Table 2**). The presence of a correlation between and NCR and TRF2 levels supports the idea that abnormal lamin A/C function, as reflected by abnormal nuclear morphology, may be causally related to the TRF2 degradation. As expected, control fibroblast cultures did not show significantly abnormal NCRs.

**Table 2:** Correlation analysis of nuclear morphological abnormality and reduction ofTRF2 within *LMNA* mutant primary fibroblast cultures.

Cell Line	PORTU8010	NORWAY1010
*LMNA* mutation	R133L	L140R
Nuclear contour ratio	0.749 ± 0.118	0.815 ± 0.074
Relative TRF2 intensity	54.6 ± 6.0	51.5 ± 5.3
Pearson’s correlation coefficient (*r* )	0.365	0.379
*P* value	0.003	0.01
Number of cells examined	65	40

*Nuclear contour ratios (NCR) were calculated as 4πA/P^*2*^, where P = Perimeter, A = Area. NCR value = 1, indicates a perfect circle. The correlation between NCR and TRF2 protein amount in cells was determined using Pearson’s correlation analysis. Pearson’s correlation coefficient (*r)* is a number between -1 and 1.* r* closer to 1 indicates a positive correlation, whereas *r* closer to -1 indicates a negative correlation.*

### INTRANUCLEAR LOCALIZATION OF TRF2 PROTEIN IN *LMNA* MUTANT CELLS

Next, we assessed the intranuclear localization of TRF2 in relation to telomeres using immuno-FISH. Co-localization analysis of TRF2 and telomeres revealed that the fraction of signals of telomeric foci that overlapped with signals of TRF2 foci was slightly less in *LMNA* mutant fibroblasts 66% in R133L and -68% in L140R as compared to 78% in the control, 82-6 (**Figures 2B,C**). Approximately 34% (*P *= 0.06) of telomeric foci in the R133L mutant and 32% (*P *= 0.174) in the L140R mutant were free of TRF2. These results agree with our western results, which showed a reduction of TRF2 protein in mass cultures. The TRF2 proteins in *LMNA* mutants, albeit reduced in amount, were co-localized to the telomeres (**Figure 2B**). These findings are consistent with the hypothesis that *LMNA* mutant fibroblasts in young passages might have lost optimum levels of shelterin complexes prior to telomere shortening, and that the reduced levels of TRF2 protein observed in *LMNA* mutants could be a causal step rather than a consequence of accelerated telomere attrition.

### R133L AND L140R MUTATIONS INDUCE ACCUMULATION OF GENOMIC DNA DAMAGE

**FIGURE 3:**
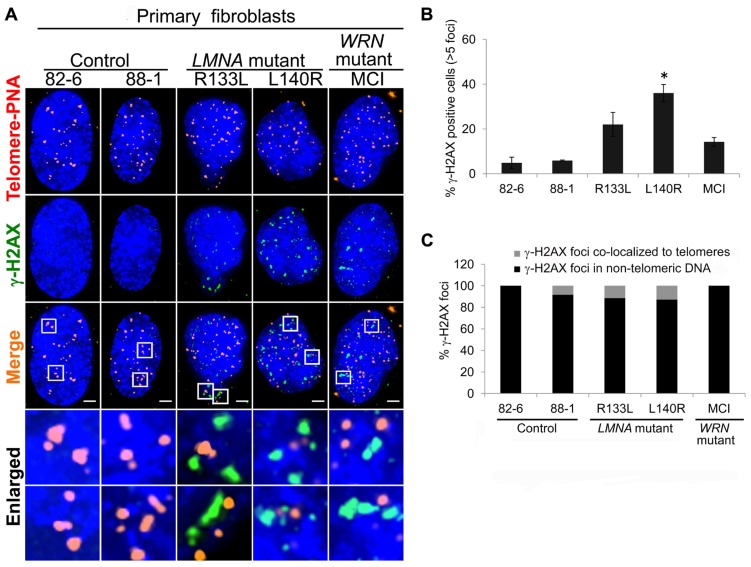
** Detection of γ-H2AX DNA damage foci in* LMNA* mutant cells.** **(A)** Immuno-FISH staining of telomeres (using a telomeric PNA probe; red), γ-H2AX (green), and DNA (using DAPI; blue) in control, *LMNA* mutant and *WRN* mutant cells. White boxes in the merged images indicate areas that are enlarged 5× and shown to the bottom. Scale bar: 2 μm. **(B)** Percentages of cells with >5 γ-H2AX-labeled foci. Data are averages of three independent experiments. Bars represent ± s.d. *P < 0.05. **(C)** Quantitation of γ-H2AX foci detected in **(A)**. Signals of γ-H2AX foci overlapping telomeres (gray) and those not overlapping (black) are shown separately. A minimum of 70 cells were analyzed for each cell line. Scale bar: 2 μm.

It has been reported that progerin induces a DNA damage response at the telomeres (Benson et al., 2010). We asked whether non-progerin producing *LMNA *mutations, R133L and L140R, also cause the accumulation of DNA damage and if so, where. To answer these questions, we assayed a marker of DNA damage, γ-H2AX foci. In control fibroblasts, up to approximately 4% of cells were positive for >5 γ-H2AX foci (**Figures 3A,B**). The fraction of cells with >5 γ-H2AX foci was increased to 22% (*P *= 0.06) in R133L and 36% (*P *= 0.01) in L140R mutants (**Figure 3B**).

To assess the degree to which the DNA damage foci in young passages of *LMNA* mutant cells were localized to the telomeres, we analyzed the co-localization of γ-H2AX and telomeres using immuno-FISH (**Figures 3A,C**). In control cells, γ-H2AX signals were hardly visible as foci at standard exposures whereas in *LMNA* mutant cells γ-H2AX foci tended to be larger than those seen in the controls (**Figure 3A**). The majority of the γ-H2AX foci present in *LMNA* mutant cells were not co-localized to telomeric DNA (89% in RI33L and 87% in L140R; **Figure 3C**). Telomeric γ-H2AX represented relatively minor fractions of the total foci. These data indicate that, during early passages, R133L and L140R *LMNA* mutant fibroblasts may sustain non-telomeric genomic damage prior to the accumulation of damage at the telomeres.

### TELOMERE STABILIZATION DOES NOT PREVENT REDUCTION OF SHELTERIN EXPRESSION IN *LMNA* MUTANTS

**FIGURE 4:**
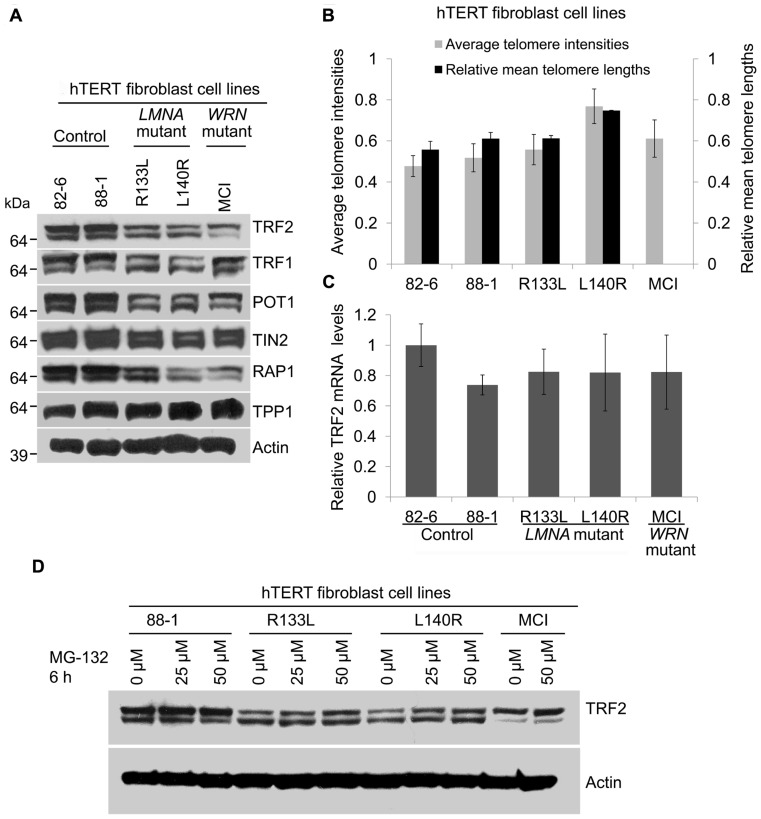
** Expression of shelterin subunits in hTERT-immortalized cell lines of control, *LMNA* mutant and*WRN* mutant fibroblasts.** **(A)** Western analysis of TRF2, TRF1, POT1, TIN2, hRap1, and TPP1 in hTERT-immortalized fibroblasts of indicated cell lines. Autoradiographies with higher exposures are shown for clarification of bands. **(B)** Analysis of relative telomere lengths expressed as average telomere intensities ± s.d. in indicated cell types, as shown in **Figure 1**. A minimum of 70 cells were analyzed for each cell lines. Relative mean telomere lengths (±s.d.) were measured in hTERT-immortalized fibroblasts using qPCR. **(C)** *TRF2* mRNA expression in hTERT-immortalized normal, *LMNA *mutant and *WRN* mutant fibroblasts. *TRF2* mRNA expression was detected by qRT-PCR. *GAPDH* mRNA was used as an internal standard. Data are presented as mean ± s.d. from three independent experiments. The control (82-6) value was taken as onefold. **(D)** Expression of TRF2 protein after proteasome inhibition in *LMNA* mutant cells. hTERT-immortalized cell lines of control and *LMNA* mutant fibroblasts were incubated in the presence or absence of MG-132 for 6 h and TRF2 protein levels were detected using immunoblotting.

To further confirm that the reduction of shelterin complex proteins and the accumulation of DNA damage seen in primary *LMNA* mutant fibroblasts was not due to shortened telomeres, we analyzed TRF2 and other shelterin protein expressions in *LMNA* mutant and control fibroblasts immortalized with hTERT. Telomerase activity in all hTERT-immortalized cell lines was confirmed with a TRAP assay (data not shown). Western analysis showed an overall reduction of most of the shelterin subunit proteins in mutant hTERT lines in a pattern similar to what was observed in primary fibroblasts; TRF2, TRF1, POT1, TIN2, and Rap1 were reduced to 54, 43, 49, 79, and 59%, respectively, in R133L mutant and 37, 36, 47, 59, and 31%, respectively, in L140R mutant hTERT fibroblasts cell lines, as compared to control hTERT lines (**Figure 4A**; **Table 1**). Both Q-FISH analysis and quantitative PCR of the telomeres showed that the telomeres in *LMNA* mutant hTERT cells were not shorter than those of control hTERT lines (**Figure 4B**). These data indicate that the reduction of the shelterin complex subunits occurs without shortened telomeres in *LMNA* mutant hTERT lines, consistent with our findings in primary fibroblasts described above.

As observed in primary fibroblasts, TRF2 mRNA levels in the *LMNA* mutant hTERT lines were comparable to those of control hTERT lines, indicating that the reduction of TRF2 occurs post-transcriptionally (**Figure 4C**). In normal human diploid fibroblasts TRF2 undergoes rapid proteasome-mediated degradation after dissociation from the telomeres during replicative senescence (Zhou et al., 2009; Fujita et al., 2010). When proteasome degradation was inhibited by MG-132, TRF2 protein levels in R133L and L140R mutants were partially restored from 49 to 67% and 33 to 63%, respectively (**Figure 4D**). This suggests that a part of the post-translational degradation of TRF2 in *LMNA* mutants is through proteasomal degradation. These data indicate that the stabilization of telomeres by hTERT does not prevent the degradation of TRF2 and that there may be other mechanisms that initiate TRF2 reduction in *LMNA* mutant fibroblasts.

### *LMNA* MUTANT hTERT FIBROBLASTS ACCUMULATE NON-TELOMERIC GENOMIC DAMAGES

**FIGURE 5:**
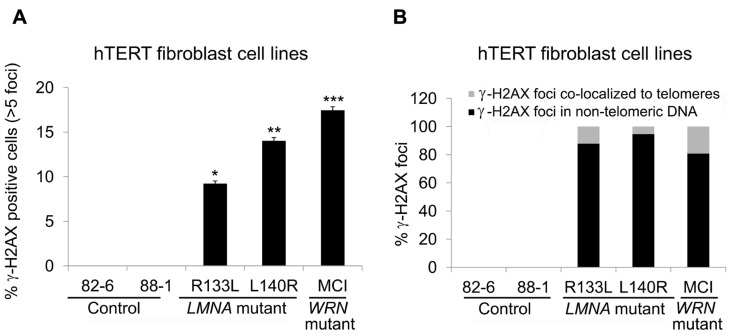
** Detection of γ-H2AX foci in control and *LMNA* mutant fibroblasts expressing hTERT.** **(A)** Percentage of cells with >5 γ-H2AX-labeled foci, as stated in **Figure 3**. *P < 0.05, **P < 0.005, ***P < 0.001. **(B)** Quantitation of γ-H2AX foci in indicated cell lines. Signals co-localizing TRF2 (gray) and those not co-localizing (black) are separately shown. In each condition approximately 70 cells were analyzed for each cell lines.

To examine whether DNA damage is involved in the reduction of TRF2 in *LMNA* mutant cells with stable telomeres, we analyzed DNA damage foci in hTERT-immortalized *LMNA* mutant and control fibroblasts. The results showed that the fraction of cells positive for >5 γ-H2AX foci in *LMNA* mutant hTERT fibroblasts was decreased compared to the *LMNA* mutant primary fibroblasts. However, the number remained higher (9 and 14% in R133L and L140R mutants, respectively) when compared to the control hTERT cell lines, which were devoid of cells with >5 γ-H2AX foci (**Figure 5A**). Co-localization analysis revealed that only 12 and 5% of total γ-H2AX foci co-localized with TRF2 (a marker of telomeres) in R133L and L140R mutant cells, respectively, whereas the rest of the DNA damage signals were distributed to non-telomeric DNA (**Figure 5B**). These results indicate that mutant lamin A/C causes the accumulation of non-telomeric DNA damage and the reduction of TRF2 in hTERT-immortalized fibroblasts without substantial telomeric damage, as observed in young primary fibroblasts.

## DISCUSSION

In this study we investigated the role of mutant lamin A in accelerating telomere shortening using *LMNA *mutant fibroblasts derived from AWS patients. We observed that the levels of TRF2, along with most of the other subunits of the shelterin complex, were significantly decreased in *LMNA* mutant fibroblast cells. TRF2 mRNA levels remained unchanged as compared to control cells with matched BrdU labeling indices. It should be emphasized that both mutant and control primary cells used in these experiments were in early passages, as evidenced by the labeling indices and the comparable lengths of the telomeres. This indicates that the reduction of TRF2 and other shelterin protein levels occurred prior to the shortening of the telomeres in *LMNA* mutant cells. The depletion in shelterin subunit protein levels in *LMNA* mutant primary fibroblasts was not corrected by introducing hTERT expression, but it was partially restored in response to proteasome inhibitor treatment. This supports our interpretation that the reduction of TRF2 in *LMNA* mutant fibroblasts is not merely secondary to the shortening of telomeres, but is primarily caused by *LMNA* mutations which lead to degradation of the TRF2 protein. In primary *LMNA* mutant cells, telomere damage and shortening contribute to further reduction of TRF2. In *LMNA* mutant cell cultures, the cells with more misshapen nuclei, as measured via NCR, exhibited lower TRF2 protein levels. This also suggests a causative role for mutant lamin A in the reduction of the TRF2 protein. In addition, *LMNA* mutant cells accumulated significant genomic DNA damage which persisted after ectopic expression of hTERT.

**FIGURE 6:**
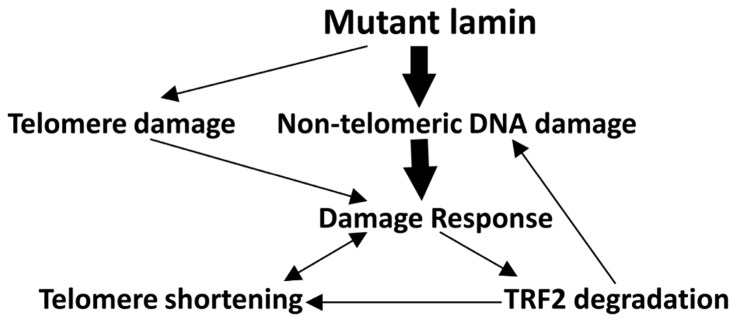
** Potential mechanisms ofTRF2 degradation and telomere shortening in *LMNA* mutant AWS cells.** Our data support the hypothesis that in young passage cells, though mutant lamin may initiate some DNA damage to the telomeres, the bulk of the DNA damage accumulates within non-telomeric DNA (bold arrows). The latter may be largely responsible for the initiation of a DNA damage response, followed by TRF2 degradation and destabilization of the telomere–shelterin complex. These alterations in turn cause rapid telomere attrition (Oh et al., 2003) and replicative senescence in these cells. Depletion of TRF2 protein along with insufficient DNA damage repair may also increase the overall levels of genomic DNA damage via a positive feedback loop (Mao et al., 2007). In senescent *LMNA *mutant cells, all the pathways may be activated.

Based on the above findings, we propose that *LMNA* mutations first induce the accumulation of double stranded DNA damage. This damage then leads to the activation of the p53 pathway, TRF2 degradation and telomere shortening (**Figure 6**). In this scenario, double stranded DNA damage accumulates in *LMNA* mutant cells in either telomeric or non-telomeric regions of the DNA, which in turn causes TRF2 degradation. The disruption of proper shelterin complex formation due to suboptimum levels of TRF2 and other shelterin components may then destabilize the telomeres, making them more vulnerable to nucleolytic degradation and accelerate telomere shortening and cellular senescence (Sfeir and de Lange, 2012). Our results showing the presence of accumulated DNA damage in both *LMNA* mutant primary and hTERT fibroblasts are consistent with such a model (**Figure 6**, bold arrows).

Considering the size of telomeric DNA relative to that of the total genome, the number of double stranded DNA damage foci per unit of DNA sequence may be higher in telomeric DNA than in non-telomeric regions in primary* LMNA* mutant cells. However, there are substantially more DNA lesions in non-telomeric DNA, consistent with a major and, perhaps, primary impact on overall genomic instability. We speculate that the decrease in shelterin complex proteins might lead to the accumulation of damage at telomeres at later passages of primary cultures correlated with increased accumulation of mutant lamin A. TRF2 is also known to have an essential role in non-telomeric DNA damage repair, namely homologous recombination (Mao et al., 2007). It is therefore, possible, that depletion of TRF2 in *LMNA* mutant AWS cells may contribute to compromised double strand break repair and additional accumulation of DNA damage (**Figure 6**). The expression levels of the shelterin subunits were also reduced in *WRN* mutant fibroblasts. This can be explained, in part, by the accumulation of genomic DNA damage.

It is not known if the pattern or degree of DNA damage is different depending on the type of *LMNA* mutation. The presence of accumulated DNA damage in our *LMNA* mutant hTERT lines is in agreement with a previous study of progerin expressing cells by Scaffidi and Misteli (2006). In contrast, Benson et al. (2010), described the accumulation of DNA damage almost exclusively at the telomeres in HGPS cells, but not in hTERT HGPS cells. Additional research is required to explain these discrepant results.

The p53–Siah1–TRF2 pathway was recently demonstrated to be a mechanism of TRF2 degradation in senescent normal fibroblasts with short telomeres (Fujita et al., 2010). Studies of doxorubicin treated colon cancer cells and lymphoblast cells provided evidence that non-telomeric DNA damage can also activate the p53-Siah1 pathway and result in proteosomal degradation of TRF2 (Fujita et al., 2010). Our observations of a preponderance of DNA damage in the non-telomeric genomic DNA of primary and *LMNA* mutant hTERT cells raise the possibility that this is the trigger leading to the activation of the p53 pathway and the degradation of TRF2 protein in *LMNA* mutant cells. We examined baseline phosphorylated p53 protein levels by western analysis (data not shown) which showed that variations among controls were larger than those between controls and patient cell lines. Therefore we were unable to determine whether accumulation of non-telomeric DNA damage in *LMNA* mutant cells involves the p53–Siah1 pathways. We observed only a partial stabilization of TRF2 by the addition of proteosomal degradation inhibitor, raising the possibility that other mechanisms of protein degradation may be contributing to the reduction of TRF2 protein in *LMNA* mutant cells. For example, DNA damage signaling cascades such as p53 and ATM induce autophagy (Hasty et al., 2013). Autophagic activity measured by the LC3-II levels was increased in skeletal muscles and other tissues derived from the *Zmpste24*^-/-^, *XPF*^-/-^, and *CSB/XPA*^-/-^ progeroid mice (Marino et al., 2008). We recently observed increased basal autophagy in *LMNA* mutant (R133L) fibroblasts used in this study (unpublished data).

We attempted to determine the TRF2 protein half-life using a protein synthesis inhibitor, cycloheximide, in hTERT-immortalized fibroblasts. The previously reported TRF2 protein half-life was longer than 12 h (Nijjar et al., 2005). Our results also indicated that the half-life of TRF2 was greater than 12 h in both control and *LMNA* mutant cells (data not shown). We were therefore unable to determine the difference between the protein half-life in *LMNA* mutant cells as compared to controls. It therefore remains a formal possibility that the reduction of TRF2 in *LMNA* mutants, at least in part, may be due to a mechanism other than protein instability, such as a decrease in translational efficacy.

Although we have only studied the association of TRF2 with telomeres, there is also a possibility that the binding of other proteins involved in telomere replication or DNA repair factors might be inhibited in *LMNA* mutant cells, thus contributing to accelerated telomere shortening. The nuclear lamina has been shown to function as a scaffold for chromatin (Broers et al., 2006; Dechat et al., 2008) and various protein complexes (Zastrow et al., 2004) involved in the structural maintenance of the nuclear membrane, gene regulation and signal transduction. In *LMNA *mutant cells, the disruption of lamina-protein containing complexes and alterations of telomere chromatin structures can affect the accessibility of telomere binding proteins, which could lead to defects in telomere maintenance, including repair and replication of telomeric DNA. Taken together, our data suggest that the decreased level of the shelterin proteins is secondary to the accumulation of DNA damage. The loss of protection of the telomeres can therefore be considered one of the causative factors for the rapid telomere attrition observed in *LMNA* mutant cells.

There is an increasing amount of evidence for DNA damage and sustained genomic instability as the central cause for laminopathies, including progeroid syndromes such as HGPS (Liu et al., 2005; Scaffidi and Misteli, 2008; Benson et al., 2010; Gonzalez-Suarez and Gonzalo, 2010). Complete loss of lamin A or mutant lamina may directly lead to genomic instability, including instability of the telomeres (Liu et al., 2005; Gonzalez-Suarez et al., 2009). Telomere dysfunction then leads to genomic instability, which triggers cellular senescence or apoptosis (Blackburn, 2001; de Lange, 2005). Our study also supports this model, suggesting that unrepaired DNA damage and therefore genomic instability is the common mechanistic cause of the pathophysiology of laminopathies.

## Conflict of Interest Statement

The authors declare that the research was conducted in the absence of any commercial or financial relationships that could be construed as a potential conflict of interest.
